# A novel prognostic model based on pyroptosis-related genes for multiple myeloma

**DOI:** 10.1186/s12920-023-01455-5

**Published:** 2023-02-23

**Authors:** Cuiling Zhang, Sungui Wu, Bing Chen

**Affiliations:** 1 Department of Hematology, Affiliated Drum Tower Hospital, Medical School of Nanjing University, 210008 Nanjing, People’s Republic of China; 2 Nanjing Drum Tower Hospital Clinical College of Nanjing University of Chinese Medicine, 210023 Nanjing, People’s Republic of China

**Keywords:** Multiple myeloma, Pyroptosis, Prognosis, bioinformatics

## Abstract

**Background:**

Multiple myeloma (MM) is an incurable and relapse-prone disease with apparently prognostic heterogeneity. At present, the risk stratification of myeloma is still incomplete. Pyroptosis, a type of programmed cell death, has been shown to regulate tumor growth and may have potential prognostic value. However, the role of pyroptosis-related genes (PRGs) in MM remains undetermined. The aims of this study were to identify potential prognostic biomarkers and to construct a predictive model related to PRGs.

**Methods:**

Sequencing and clinical data were obtained from The Cancer Genome Atlas (TCGA) and Gene Expression Omnibus (GEO) databases. Non-negative matrix factorization (NMF) was performed to identify molecular subtype screening. LASSO regression was used to screen for prognostic markers, and then a risk score model was constructed. The Maxstat package was utilized to calculate the optimal cutoff value, according to which patients were divided into a high-risk group and a low-risk group, and the survival curves were plotted using the Kaplan-Meier (K-M) method. Nomograms and calibration curves were established using the rms package.

**Results:**

A total of 33 PRGs were extracted from the TCGA database underlying which 4 MM molecular subtypes were defined. Patients in cluster 1 had poorer survival than those in cluster 2 (*p* = 0.035). A total of 9 PRGs were screened out as prognostic markers, and the predictive ability of the 9-gene risk score for 3-year survival was best (AUC = 0.658). Patients in the high-risk group had worse survival than those in the low-risk group (*p* < 0.001), which was consistent with the results verified by the GSE2658 dataset. The nomogram constructed by gender, age, International Staging System (ISS) stage, and risk score had the best prognostic predictive performance with a c-index of 0.721.

**Conclusion:**

Our model could enhance the predictive ability of ISS staging and give a reference for clinical decision-making. The new, prognostic, and pyroptosis-related markers screened out by us may facilitate the development of novel risk stratification for MM.

**Clinical trial registration:**

Not applicable.

**Supplementary Information:**

The online version contains supplementary material available at 10.1186/s12920-023-01455-5.

## Introduction

Multiple myeloma (MM) is a malignant neoplasm of plasma cells, accounting for 1% of neoplastic diseases and ranking second among hematological malignancies [1]. In recent years, with the research and application of new drugs, the prognosis of MM has been significantly improved. Despite these advances, MM remains an incurable disease, and most patients eventually relapse [2]. According to statistics, approximately 100,000 patients die from MM every year worldwide [3]. To predict patient prognoses more precisely and make an optimal therapy decision, accurate risk stratification is important, while current MM staging systems lack sensitivity and specificity in a proportion of patients, and uncovering other prognostic factors is of great significance [4, 5].

Pyroptosis is a type of programmed cell death, manifested by the phenomenon of cellular swelling to membrane rupture, resulting in the release of cellular contents and inducing strong inflammatory responses [6]. It has been shown to be associated with a variety of tumors, including hematological malignancies, and its role on tumors is dual, which means it can both promote and inhibit tumor growth [7, 8]. Some of its molecular components have been shown to regulate tumor proliferation, metastasis, therapeutic resistance, and antitumor immunity, making them correlated with survival of patients and available for predicting prognoses [9].

At present, there are few studies on pyroptosis in MM [10–13]. Xia et al. [10] found that PRMT5 regulates pyroptosis in MM. Gaikwad et al. [11] found that a small molecule stabilizer of the MYC G4-quadruplex induces endoplasmic reticulum pyroptosis in MM. Wang et al. [12] constructed a prognostic model including a risk score with 11 pyroptosis-related genes (PRGs) from the GSE136324 dataset, but the small sample size of the verification cohorts might affect the reliability of the results. Li et al. [13] constructed a prognostic gene model based on 6 PRGs from the GSE24080 dataset, but they didn’t assess the mutation characteristics of PRGs. Moreover, there is a high false-positive rate in the single dataset analysis. Different datasets and different microarray platforms may yield different results. To identify more reliable and robust novel prognostic markers and overcome these inconsistencies, further analysis using different datasets is needed.

Hence, the aims of this study were (1) to explore the expression and mutation characteristics as well as immune correlations of PRGs in MM, (2) to determine distinct pyroptosis patterns based on the expression of PRGs and classify the patients, (3) to identify potential prognostic biomarkers and construct a predictive model related to PRGs and (4) to verify the reliability of these results based on The Cancer Genome Atlas (TCGA) and Gene Expression Omnibus (GEO) databases. Our study further identified the role of PRGs in the prognosis of MM, providing some insights for follow-up studies.

## Materials and methods

### Datasets and preprocessing

The transcriptome sequencing (RNA-seq) data and the whole exome sequencing (WES) data of CD 138^+^ myeloma cells within bone marrow from 764 patients, along with corresponding clinical characteristics and follow-up data, were obtained from the MMRF-COMPASS project in the TCGA database (https://portal.gdc.cancer.gov/projects/MMRF-COMMPASS, accession number: MMRF-COMMPASS, accessed date: 1st June 2022). The gene expression microarray results and the annotation files of the GSE2658 dataset (microarray platform: GPL570 [HG-U133_Plus_2] Affymetrix Human Genome U133 Plus 2.0 Array) [13] and the GSE39754 dataset (microarray platform: GPL5175 [HuEx-1_0-st] Affymetrix Human Exon 1.0 ST Array) [14] were downloaded from the GEO database (https://www.ncbi.nlm.nih.gov/geo/query/acc.cgi?acc=GSE2658, accession number: GSE2658, https://www.ncbi.nlm.nih.gov/geo/query/acc.cgi?acc=GSE39754, accession number: GSE39754, accessed date: 1st June 2022). The GSE2658 dataset [14], which contained transcriptomic data of CD138^+^ myeloma cells from 559 patients and matched prognostic information, was used as a validation set for this study. The GSE39754 dataset [15], which contained transcriptome data of CD138^+^ myeloma cells from 170 patients and plasma cells from 6 normal donors, served as an analysis dataset for the differential analysis of PRGs expression and immune cell infiltration. The basic clinical characteristics of the three above-mentioned datasets were presented in Additional file 1.

### Somatic mutation, expression differences, correlation analysis and clinical correlation of PRGs

A total of 33 PRGs (Additional file 2) were extracted from the RNA-seq data, among which the MMRF project had 33 PRGs and the GSE39754 dataset had 29 PRGs. Differentially expressed PRGs in the myeloma cells and control plasma cells from the GSE39754 dataset were identified by the Wilcoxon test with *p* < 0.05. The expression levels of these PRGs were displayed by heatmap using the pheatmap package [16]. The relationship between different PRGs from the MMRF project was analyzed using Pearson’s correlation. A total of 31 PRGs were extracted from the WES data of the MMRF project. The mutation frequency and classification of these PRGs were analyzed using the maftools package [17].

Based on the MMRF project, the differences of PRG expression within different clinical subgroups (gender, age, and International Staging System (ISS) stage) were compared by the Wilcoxon test.

### Immune infiltration analysis

The immune cell infiltration for every sample from the MMRF project and GSE39754 dataset was evaluated by the CIBERSORTx tool (https://cibersortx.stanford.edu/, accessed date: 5th June 2022) [18], and samples with *p* < 0.05 were filtered out. The Wilcoxon test was used to compare differences in the degrees of immune cell infiltration between the case and control groups from the GSE39754 dataset. Based on the MMRF project, correlation analysis was performed between each immune cell and between immune cells and PRGs. Pearson correlation coefficient was then calculated.

### NMF molecular subtype construction

Non-negative matrix factorization (NMF) is an effective technique to decompose a non-negative matrix into the product of two non-negative matrices. For any given non-negative matrix V, it can be divided into a non-negative matrix W and a non-negative matrix H to satisfy the condition V = W × H. Each column in the V matrix represents an observation point, and each row represents a feature. The W matrix is called the base matrix, and the H matrix is called the coefficient matrix or the weight matrix. By replacing the original matrix with the coefficient matrix H, the original matrix can be dimensionally reduced to obtain the matrix containing the feature set. Molecular subtype screening was performed based on the expression of PRGs from the MMRF project using the NMF package [19]. According to the degree of cophenetic value changing with K, the rank before the maximum changing point was determined to be the optimal cluster number. In addition, we analyzed the prognostic differences of patients in different clusters.

### Gene set variation analysis (GSVA)

The limma package [20] was utilized to identify differentially expressed genes (DEGs) between clusters using a linear model. The DEGs screening criteria were adj.*p* value < 0.05 and |log2FC| > 1. GSVA, a non-parametric unsupervised algorithm, can calculate enrichment scores for specific gene sets in each sample. We performed the GSVA analysis on the DEGs matrix using the GSVA package [21], and selected “c2.cp.kegg.v7.4.symbols.gmt” as the reference gene set. Moreover, the limma package was used for differential pathway screening, and *p* < 0.05 was set as the screening threshold.

### Identification of prognostic marker (LASSO analysis)

The LASSO (Least absolute shrinkage and selection operator, Tibshirani) method is a compression estimation [22]. By shrinking the regression coefficients and reducing some of them to zero, a penalty function can be constructed to obtain a more refined model. It preserves the advantages of subset shrinkage and is a biased estimation for processing data with complex collinearity. We used univariate Cox regression to screen for PRGs associated with the survival of patients with MM from the MMRF project, and the LASSO regression to screen for prognostic markers. Variable filtering was performed using the glmnet function of the glmnet package [23]. The cv.glmnet function was used for cross-validation. The combination of prognostic markers with the smallest cross-validation (CV) coefficient was then obtained.

### Risk scoring and prognostic predictive model construction

The risk score (RS) for each case was calculated as follows:

$$RS=\sum _{i=1}^{n}{Coef}_{i}\times {Exp}_{i}$$

Coef was the LASSO regression coefficient, and Exp was the RNA expression level (log2 conversion).

The survivalROC package [24] was used to analyze the predictive ability of the RS for 1, 3, and 5-year survival of patients from the MMRF project, and the predicted receiver operating characteristic (ROC) curves were plotted and the area under the curve (AUC) values were calculated. The Maxstat package [25] was used to calculate the optimal cutoff value, according to which patients from the MMRF project were divided into a high-risk group and a low-risk group, and the survival curves were plotted using the Kaplan-Meier (K-M) method.

The Cox equal proportional hazards model was established to assess the impact of other clinical characteristics, including age, gender, and ISS stage, on patient prognoses. The Forestmodel package [26] was employed to generate forest plots. And the clinical characteristics having a significant effect on prognoses were added to multivariate Cox regression as covariates to evaluate the independent predictive ability of the RS on patient prognoses, and a forest plot was drawn.

Finally, nomograms constructed with different variables and calibration curves were established using the rms package [27] to visualize the model results and to make the results of the prediction model more readable, and the consistency index (c-index) was calculated to assess the predictive power of the nomogram for survival.

### Statistical analysis

All data calculations and statistical analyses were performed using R programming (https://www.r-project.org/, version 4.2.0). The Benjamini-Hochberg (BH) procedure was used for multiple testing corrections, and a false discovery rate (FDR) correction was used to reduce the false positive rate in multiple testing. For the comparison of two groups of continuous variables, the statistical significance in normally distributed variables was estimated by the independent Student’s *t*-test, and the difference in non-normally distributed variables was analyzed by the Mann-Whitney U test (i.e., the Wilcoxon rank sum test). For the comparison of three or more groups of continuous variables, a one-way analysis of variance (ANOVA) is used to determine whether or not there was a statistically significant difference. The ROC curves were drawn using the survivalROC package, and the AUC values were calculated to assess the accuracy of the RS in estimating prognoses. All statistical *p* values were two-sided, with *p* < 0.05 being considered statistically significant.

## Results

### Landscape of PRG expression and somatic mutation in MM

**Fig. 1:**
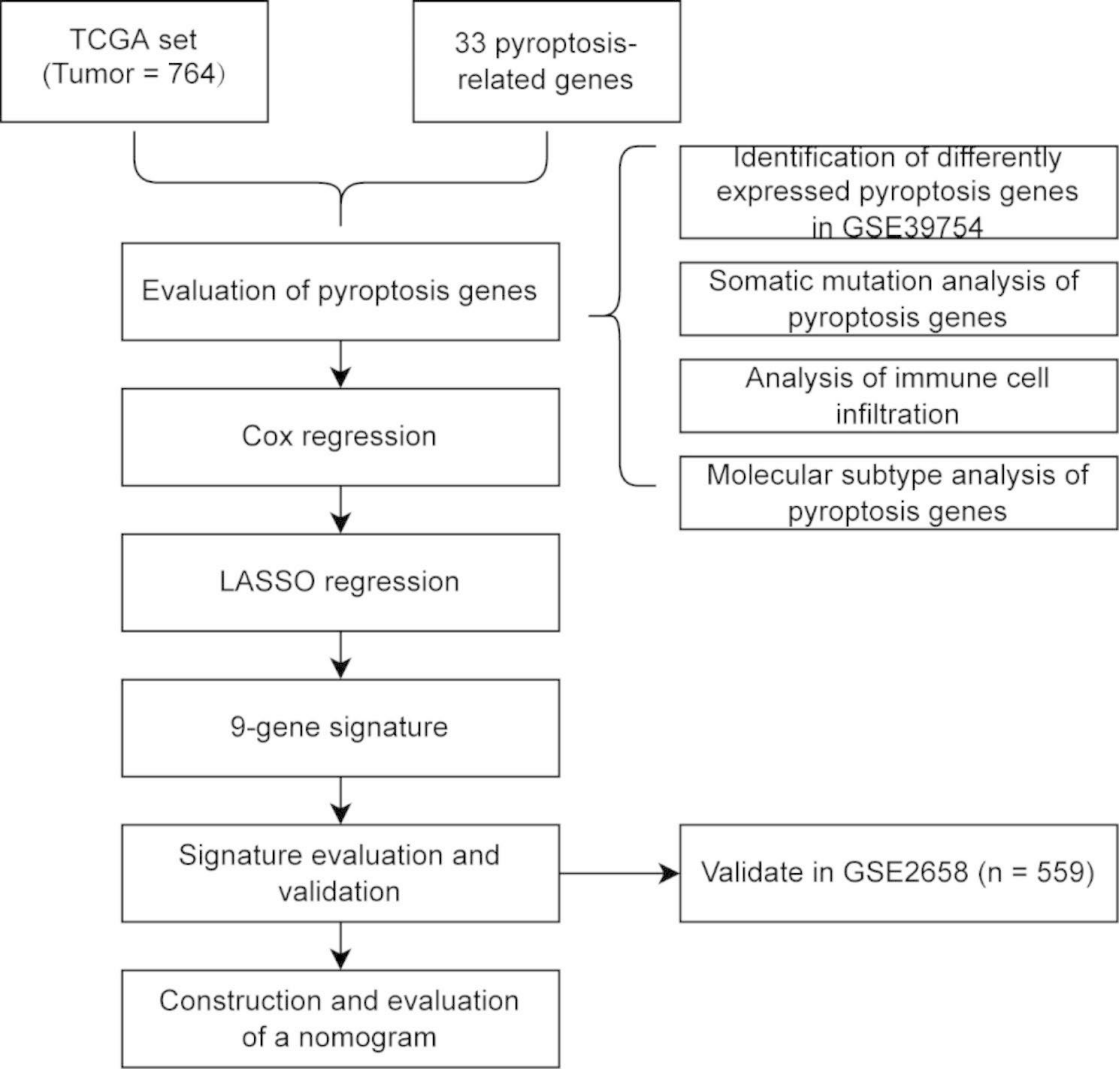
The workflow chart of this study

**Fig. 2:**
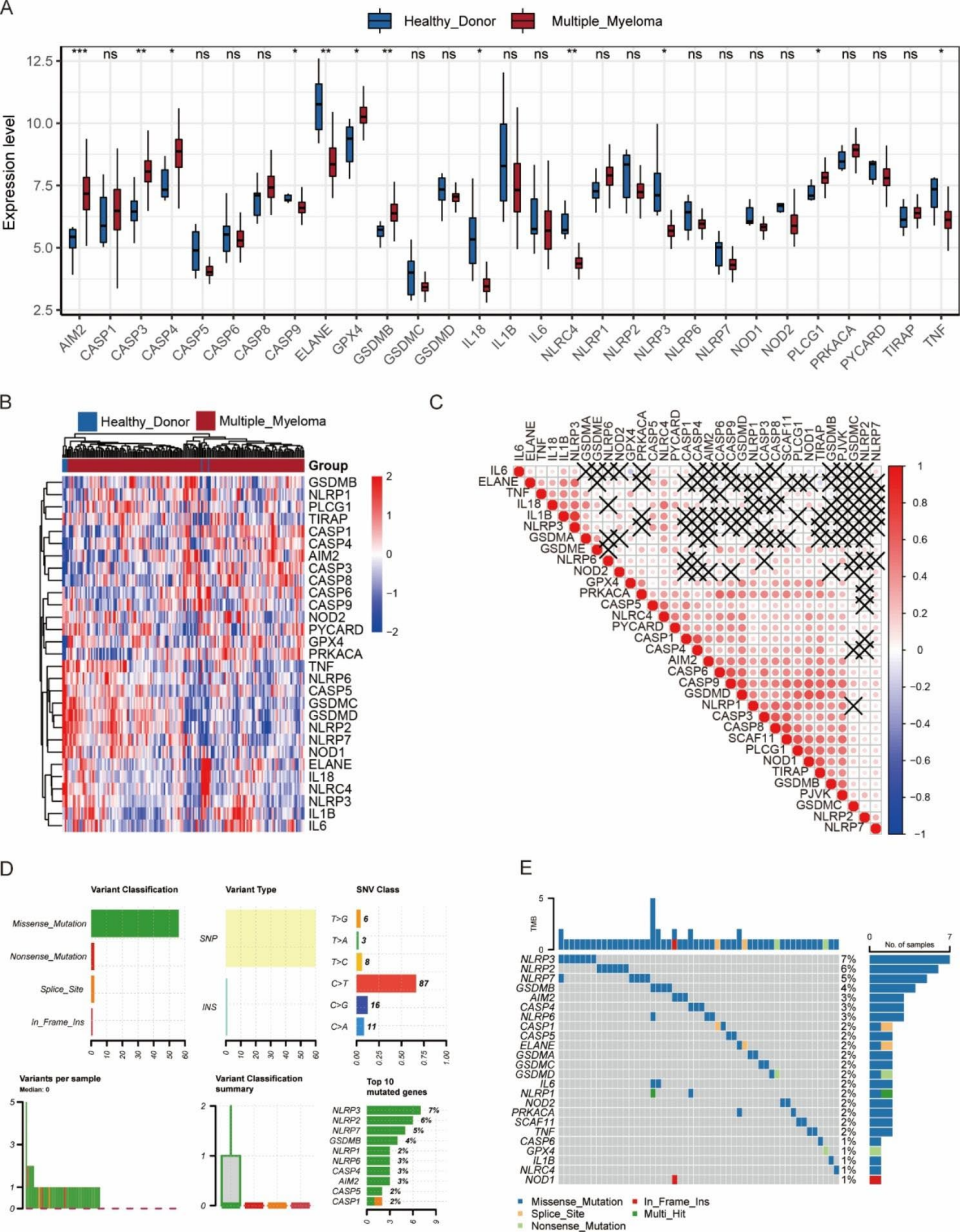
Expression and mutation of pyroptosis-related genes (PRGs). **(A)** The box plot shows expression differences of PRGs in patients with multiple myeloma and controls from the GSE39754 dataset. *: *p* < 0.05; **: *p* < 0.01; ***: *p* < 0.001; ns: not significant. **(B)** The heatmap displaying expression levels of PRGs in patients with multiple myeloma and controls from the GSE39754 dataset. **(C)** The correlation matrix for the 33 PRGs from the MMRF project. Red represents a positive correlation. Blue represents a negative correlation. The darker color represents a larger correlation index. Black X represents no statistical significance. **(D)** The somatic mutation frequency and classification of PRGs from the MMRF project. **(E)** The waterfall plot of somatic mutation of PRGs from the MMRF project

**Fig. 3:**
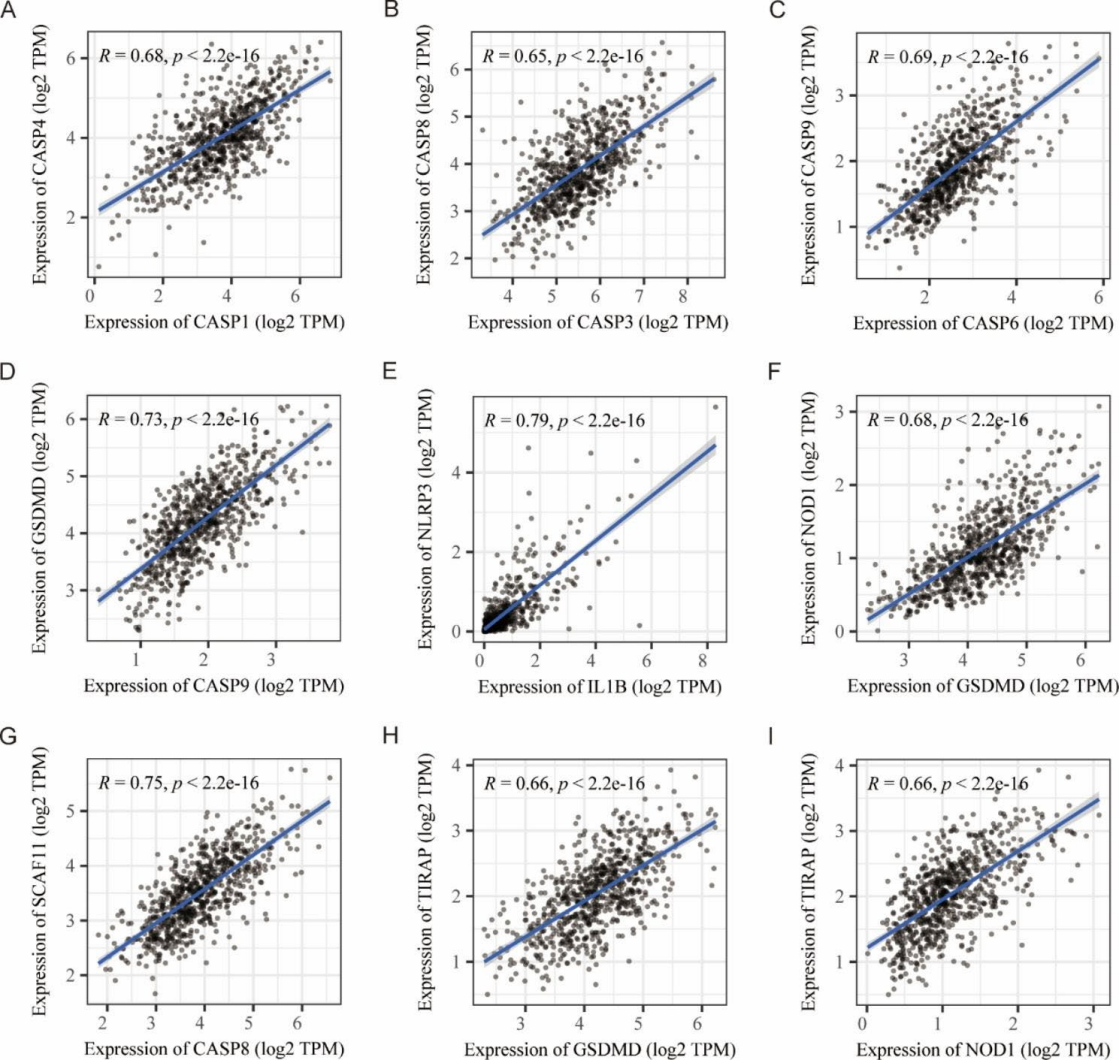
The dot-line plots of Pearson correlation analysis between each pyroptosis-related gene from the MMRF project. (A-I) Pyroptosis-related genes with a significant correlation and a correlation coefficient greater than 0.65

**Fig. 4:**
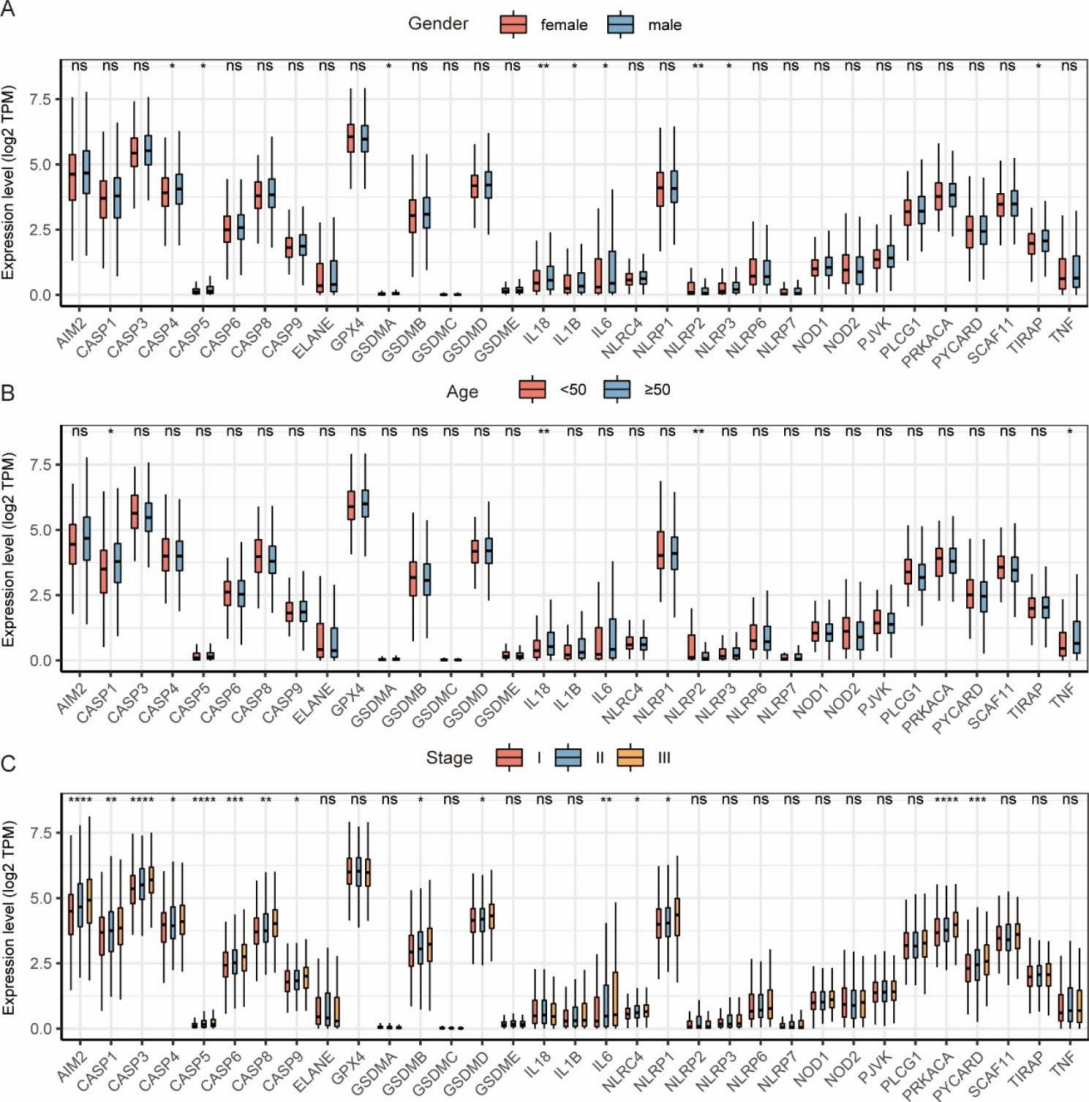
Clinical subgroup analysis of pyroptosis-related genes (PRGs) expression based on the MMRF project. **(A)** The expression of PRGs in the gender subgroup. **(B)** The expression of PRGs in the age subgroup. **(C)** The expression of PRGs in the ISS stage subgroup. *: *p* < 0.05; **: *p* < 0.01; ***: *p* < 0.001; ****: *p* < 0.0001; ns: not significant

The workflow chart was shown in Fig. 1. The expression levels of 29 PRGs in patients with MM and controls from the GSE39754 dataset were displayed as heat maps (Fig. 2B). By comparing them using the Wilcoxon test, we found that 12 PRGs were significantly differentially expressed, out of which 6 PRGs (GPX4, CASP4, PLCG1, CASP3, GSDMB, and AIM2) were upregulated in patients with MM (Fig. 2A). The gene correlation matrix for the 33 PRGs from the MMRF project was depicted in Fig. 2C, and the PRGs with Pearson correlation coefficient greater than 0.65 were shown in Fig. 3. Next, we compared the expression difference of each PRG within three clinical subgroups. In gender subgroups, CASP4, CASP5, GSDMA, IL18, IL1B, IL6, NLRP3, NLRP2, and TIRAP were significantly differentially expressed between males and females. In age subgroups, there were differences in the expression of CASP1, IL18, NLRP3, and TNF between the patients aged < 50 years and the patients aged ≥ 50 years. In ISS stage subgroups, the expression levels of genes, i.e., AIM2, CASP1, CASP3, CASP4, CASP5, CASP6, CASP8, CASP9, GSDMB, GSDMD, IL6, NLRC4, NLRP1, PRKACA, and PYCARD, were statistically different among stage I, II, and III, and the expression levels increased with the stage (Fig. 4).

The somatic mutation analysis revealed that PRG mutations were present in 14% (107/764) patient samples, and 6.8% (52/764) samples have non-synonymous mutations. Among 33 PRGs, 31 genes were found to be mutated, of which 24 genes were non-synonymously mutated. The missense mutation was the most common variant classification, and C > T was the most common single nucleotide variation (SNV). The gene with the highest mutation frequency was NLRP3, followed by NLRP2, NLRP7, GSDMB, AIM2, CASP14, and NLRP4 (Fig. 2D-E).

### Differences of immune infiltration and correlation between PRGs and immune cells

**Fig. 5:**
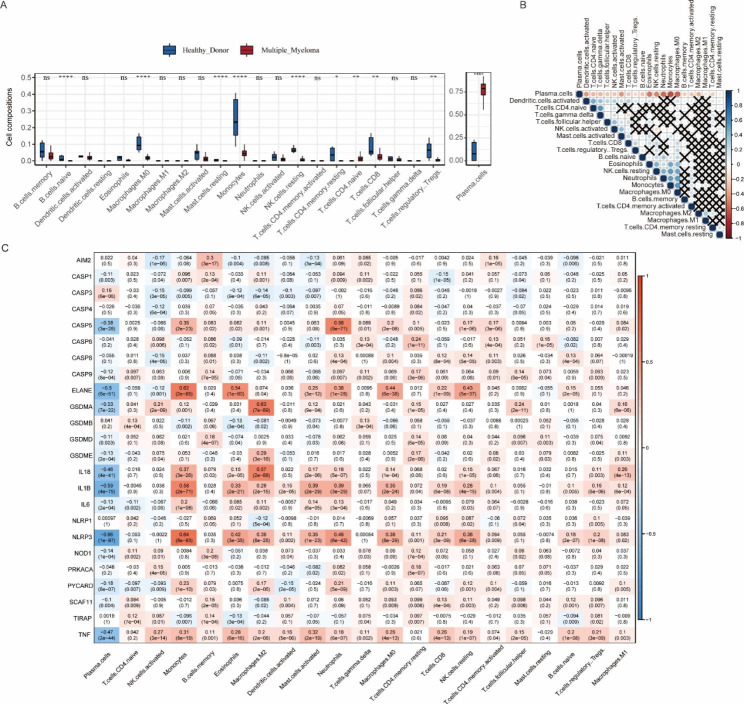
Analysis of immune infiltration. **(A)** Differences of immune infiltration between patients with multiple myeloma and controls from the GSE39754 dataset. *: *p* < 0.05; **: *p* < 0.01; ***: *p* < 0.001; ****: *p* < 0.0001; ns: not significant. **(B)** The matrix of the correlations between the infiltration degrees of each immune cell in the MMRF project. Blue represents a positive correlation. Red represents a negative correlation. The darker color represents a larger correlation index. Black X represents no statistical significance. **(C)** The correlation matrix between pyroptosis-related genes and immune cells in the MMRF project. Red represents a positive correlation. Blue represents a negative correlation. The darker color represents a larger correlation index. Correlation coefficients and *p* values were marked in the box

The differences in the degree of infiltration of 22 immune cells between patients with MM and controls from the GSE39754 dataset were shown in Fig. 5A. Based on the MMRF project, we analyzed the correlations of infiltration degrees between each immune cell, and the correlation matrix was shown in Fig. 5B. Finally, we evaluated the relationship between PRGs and immune cells (Fig. 5C). The result indicated that ELANE, GSDMA, IL18, IL1B, NLRP3, and TNF were significantly negatively correlated with plasma cells; GSDMA and IL18 were significantly positively correlated with M2 macrophages; ELANE was significantly positively correlated with M0 macrophages and resting NK cells.

### Analysis of MM molecular subtypes based on PRGs

**Fig. 6:**
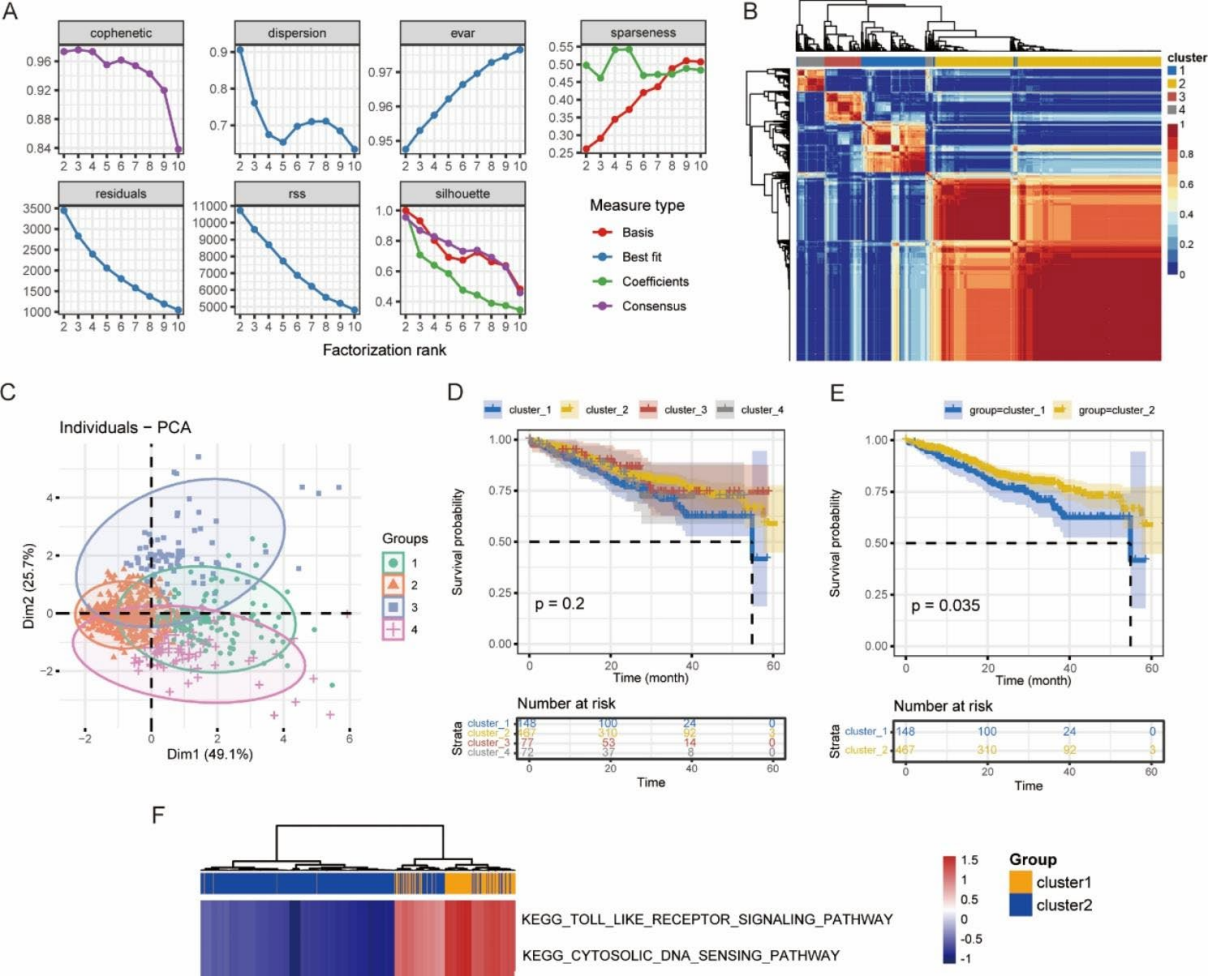
Molecular subtype analysis based on the MMRF project. **(A)** The process of NMF clustering. **(B)** The consensus map of NMF clustering. **(C)** The plot of principal component analysis. **(D)** The survival curves for 4 clusters (the K-M method). **(E)** The survival curves of cluster 1 and cluste 2 (the K-M method). **(F)** The heatmap of differential pathways in cluster 1 and cluster 2 by the GSVA analysis

**Fig. 7:**
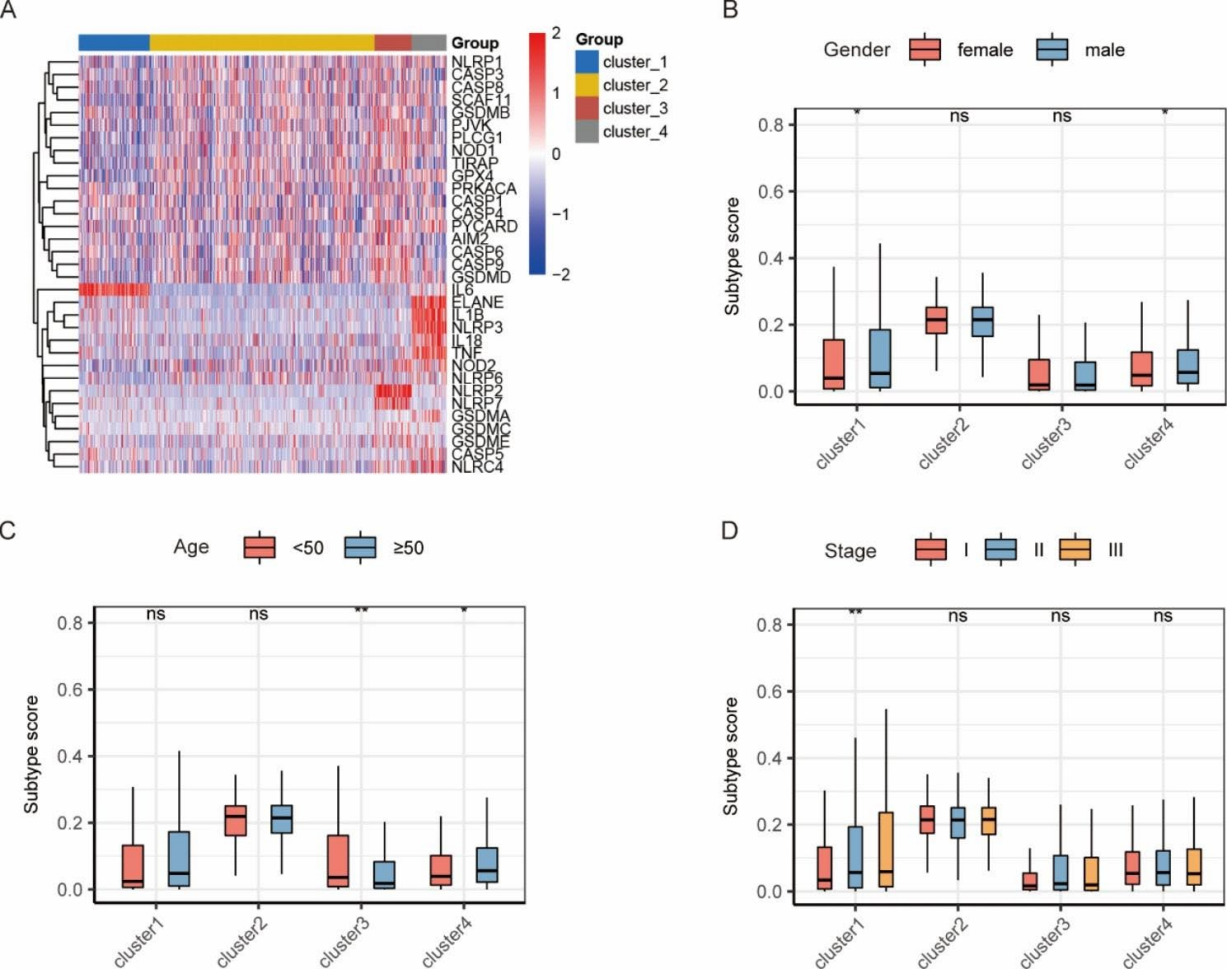
Pyroptosis-related gene expression in 4 clusters and pyroptosis scores within three clinical subgroups in each cluster based on the MMRF project. **(A)** The heatmap of pyroptosis-related gene expression in 4 clusters. The comparison of pyroptosis scores for gender **(B)**, age **(C)**, and ISS stage **(D)** subgroups in 4 clusters. *: *p* < 0.05; **: *p* < 0.01; ns: not significant

The NMF classification results showed that the cophenetic value began to drop significantly when rank = 4, so the optimal number of clusters was 4 (Fig. 6A). The consensus map of the NMF clustering and the principal component analysis plot were displayed in Fig. 6B-C, and the heatmap of the PRG expression in 4 clusters was depicted in Fig. 7A. Then we analyzed the prognoses of patients in 4 clusters by plotting the survival curve (the K-M method), and found that there was no difference in the survival time of patients in general, while multiple comparisons revealed that the survival time of patients in cluster 1 and cluster 2 was statistically different, and patients in the two clusters accounted for 80.5% (615/764) of all cases (Fig. 6D-E). The DEG analysis between the two clusters identified 372 up-regulated genes and 36 down-regulated genes. By the GSVA analysis of these DEGs, two differential pathways including the toll-like receptor signaling pathway and cytosolic DNA sensing pathway were identified (Fig. 6F). Subsequently, we compared the pyroptosis scores within three clinical subgroups in each cluster and found that males had higher scores in cluster 1 and cluster 4; patients aged < 50 years had higher scores in cluster 3, while patients aged ≥ 50 years had higher scores in cluster 4; and scores in cluster 1 increased with the ISS stage (Fig. 7B-D).

**Fig. 8:**
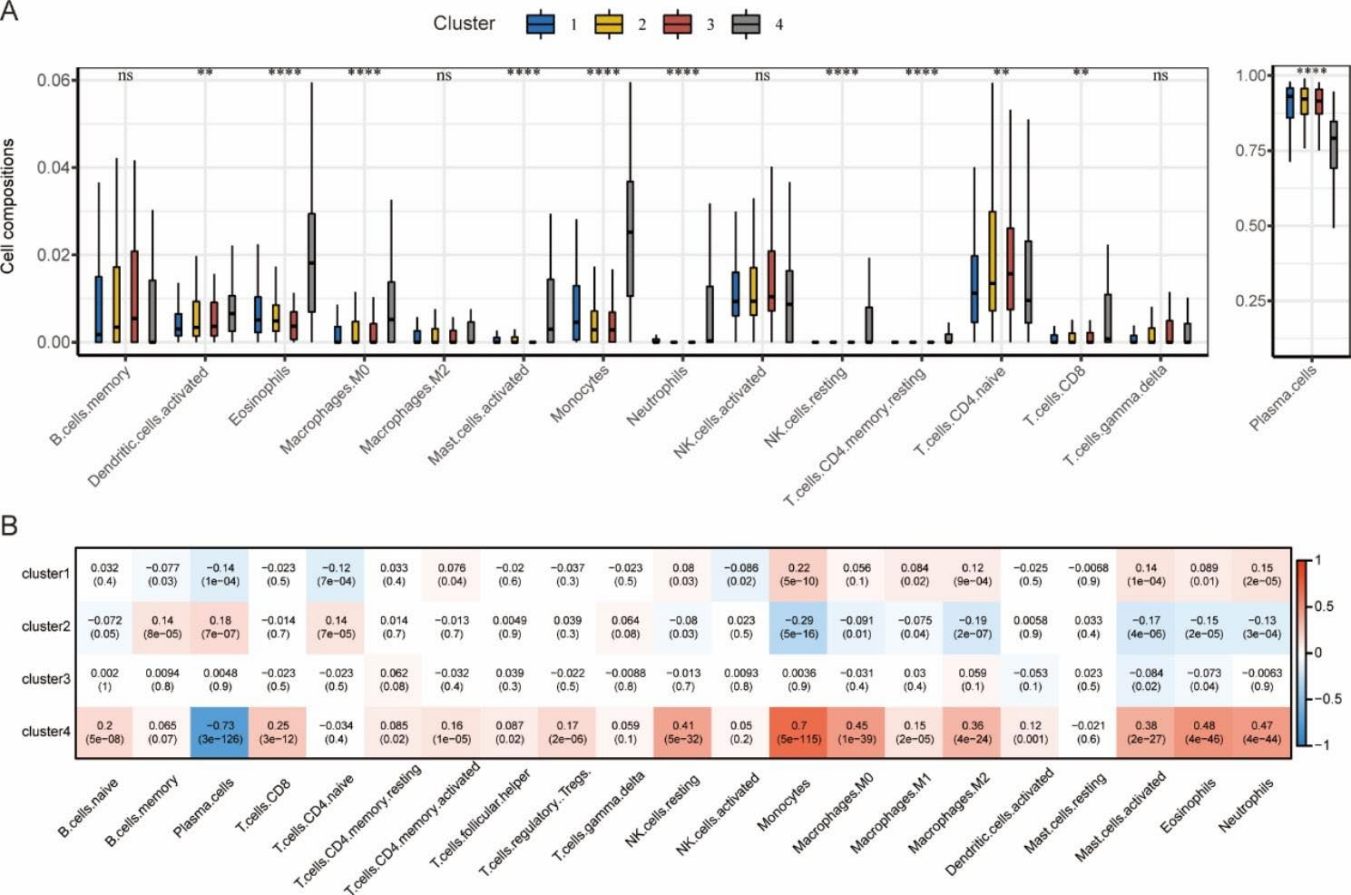
Immune infiltration in 4 clusters and the correlations between pyroptosis scores of each cluster and immune cells based on the MMRF project. **(A)** The box plots of immune infiltration in 4 clusters. **: *p* < 0.01; ****: *p* < 0.0001; ns: not significant. **(B)** The matrix of the correlations between pyroptosis scores of each cluster and immune cells. Red represents a positive correlation. Blue represents a negative correlation. The darker color represents a larger correlation index. Correlation coefficients and *p* values were marked in the box

The immune infiltration analysis of 4 clusters indicated that the infiltration degrees of multiple immune cells in cluster 4 were higher than in other clusters, while for plasma cells, which accounted for the highest proportion of all immune cells, the infiltration degree was the lowest in cluster 4 (Fig. 8A-B).

### Prognostic marker screening and RS calculation based on PRGs

**Fig. 9:**
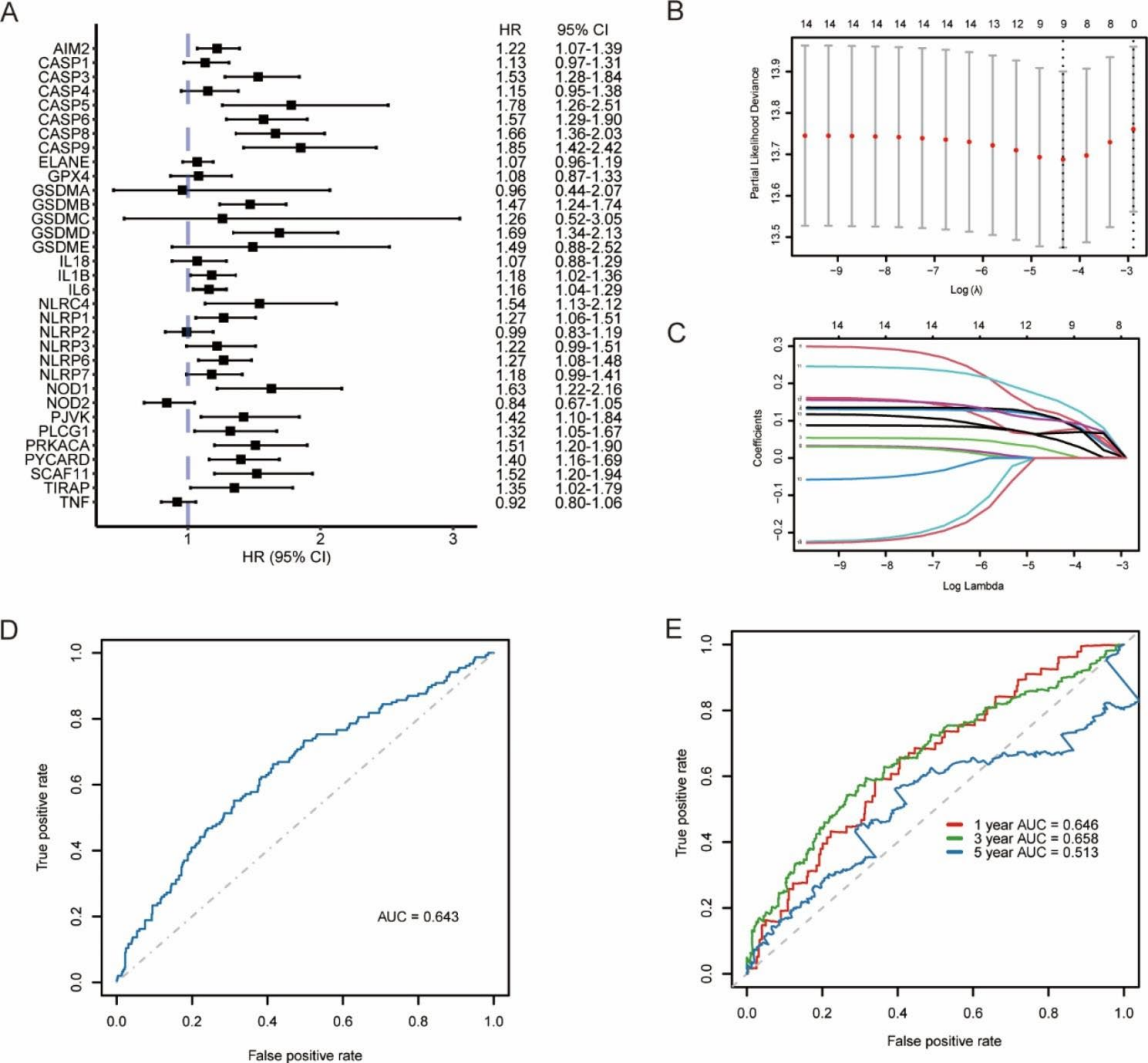
Survival analysis of pyroptosis-related genes (PRGs) and prognostic marker screening by the LASSO regression based on the MMRF project. **(A)** The forest plot of the effect of PRG expression levels on patient survival by the Cox regression analysis. **(B-C)** Use of the LASSO model to screen prognostic markers and use of the partial likelihood bias with 10-fold cross-validation to determine the optimal λ. **(D)** The ROC curve was used to evaluate the predictive ability of the LASSO model. **(E)** The ROC curves and AUC values of the risk score for predicting 1, 3, and 5-year survival of patients

The univariate Cox regression analysis results showed that 14 PRGs, i.e., AIM2, CASP5, IL1B, IL6, NLRC4, NLRP1, NLRP6, NOD1, PJVK, PLCG1, PRKACA, PYCARD, SCAF11, and TIRAP, were significantly associated with the survival of patients with MM (Fig. 9A). By the LASSO regression analysis, a total of 9 PRGs were screened out as prognostic markers, which were AIM2, CASP5, IL1B, IL6, NLRP6, NOD1, PRKACA, PYCARD, and SCAF11 respectively (Fig. 9B-C). The AUC for the LASSO regression model was 0.643, indicating a relatively good prognostic predictive ability (Fig. 9D).

**Fig. 10:**
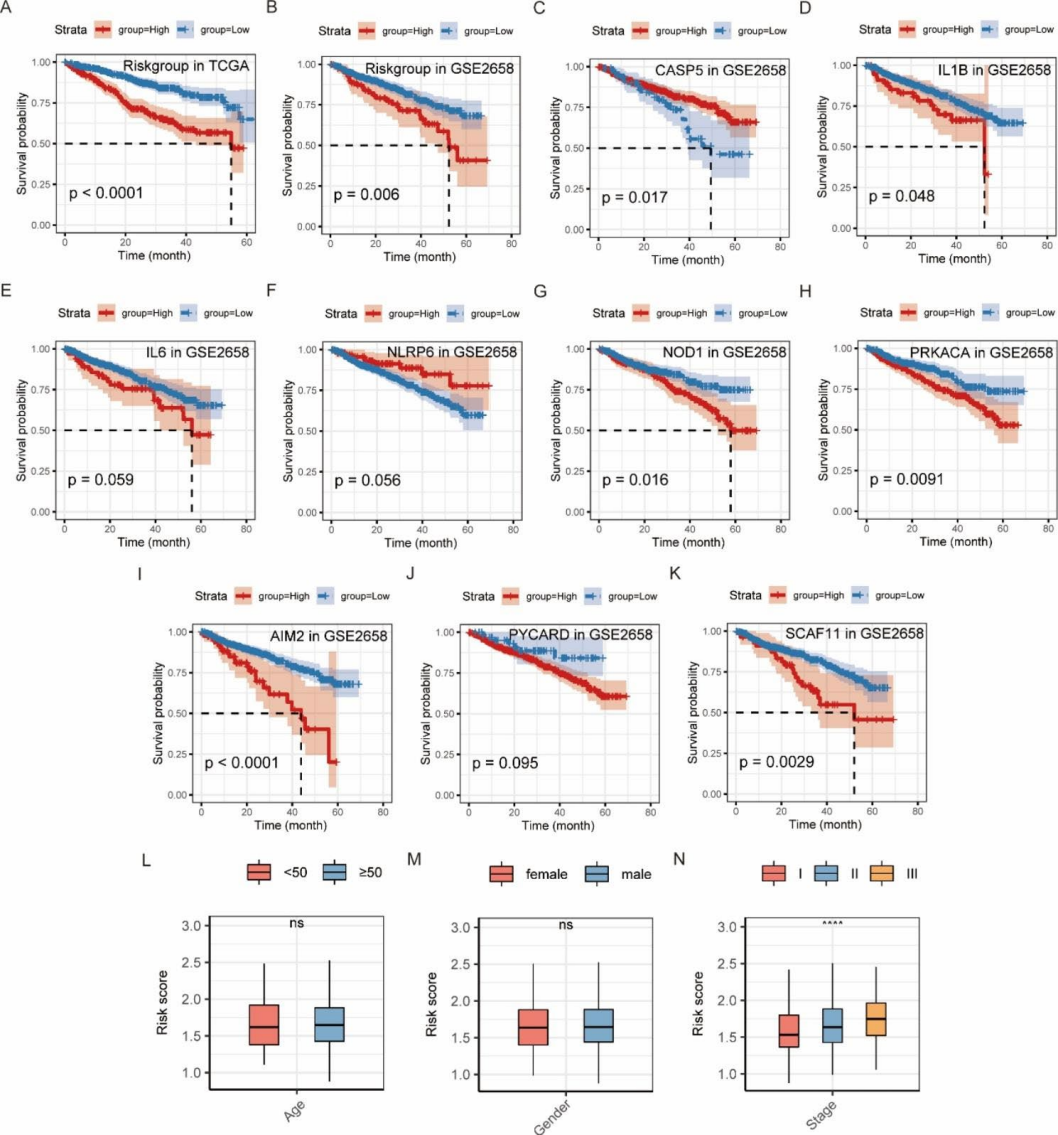
Survival analysis of patients from the MMRF project grouped by the risk score and validation by the GSE2658 dataset. **(A)** The survival curves for patients in the high- and low-risk groups from the MMRF project (the K-M method). **(B)** The survival curves for patients in the high- and low-risk groups from the GSE2658 dataset (the K-M method). **(C-K)** The survival curves for patients in high and low expression groups of 9 prognostic genes in the GSE2658 dataset (the K-M method). **(L-N)** Differences of risk scores in clinical subgroups of age, gender, and ISS stage. ****: *p* < 0.0001; ns: not significant

According to the analysis results of the LASSO regression model, the coefficients of the candidate prognostic markers were determined, and the RS was calculated as follows: RS = 0.0485 × AIM2 + 0.0735 × CASP5 + 0.0152 × IL1B + 0.1043 × IL6 + 0.1053 × NLRP6 + 0.1188 × NOD1 + 0.1533 × PRKACA + 0.1004 × PYCARD + 0.0674 × SCAF11. The AUCs of 1, 3, and 5-year survival predicted by RS were 0.646, 0.658, and 0.513, respectively (Fig. 9E). The optimal cutoff value for the RS predicting survival in patients with MM was 1.7714. According to the cutoff value, patients with MM were divided into a high-risk group and a low-risk group, and patients without survival information were excluded. The survival analysis showed that the survival time of patients in the high-risk group was significantly shorter than that in the low-risk group (Fig. 10A).

The GSE2658 dataset was used to validate the reliability of the 9-gene model, and the result was consistent with that in the MMRF project (Fig. 10B). We then performed the survival analyses for each of these genes, and found that the expression levels of genes (CASP5, IL1B, NOD1, PRKACA, AIM2, and SCAF11) were significantly related to patient survival (Fig. 10C-K).

The comparison results of RSs among ISS stage I, II, and III showed that the higher the stage, the higher the RS, and that the difference was statistically significant, while there were no differences in RSs between males and females and between the patients aged < 50 years and the patients aged ≥ 50 years (Fig. 10L-N).

#### Construction of the individualized prognostic prediction model

**Fig. 11:**
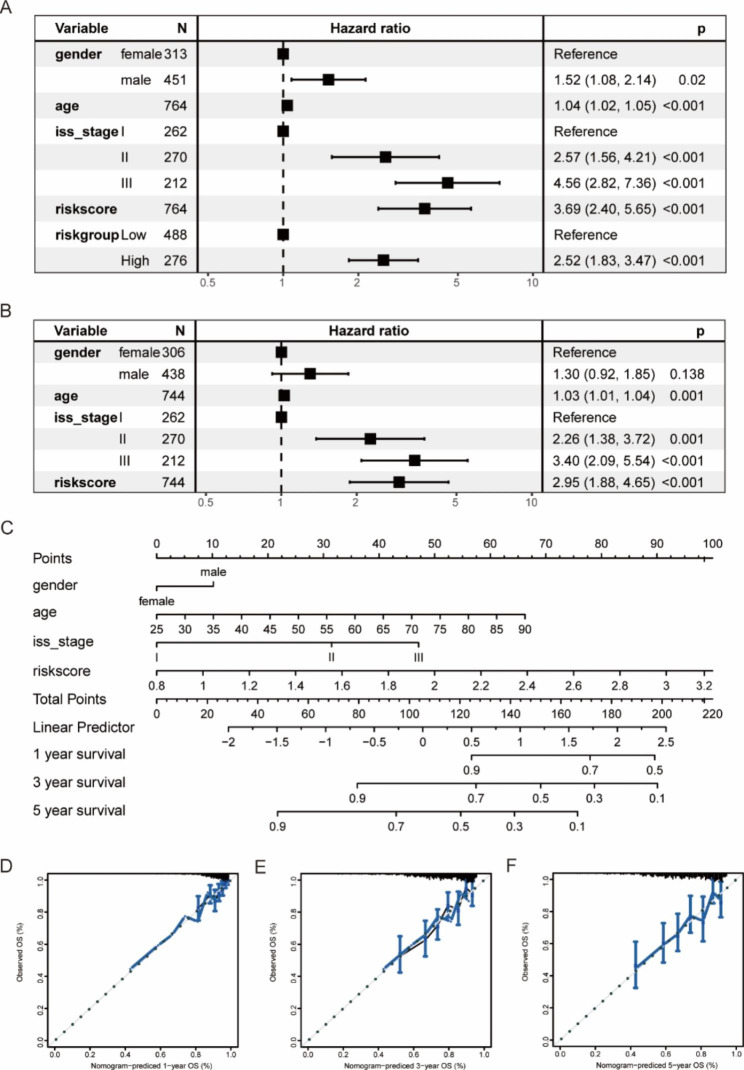
Construction of a predictive nomogram based on the MMRF project. **(A-B)** The forest plots of the effect of gender, age, ISS stage, and risk score on patient survival by the univariate and multivariate Cox regression analysis. **(C)** The nomogram was constructed by gender, age, ISS stage, and risk score. **(D-F)** The calibration curves for the nomogram predicting 1-, 3-, and 5-year survival for patients with multiple myeloma

The univariate Cox regression analysis demonstrated that in addition to RS, the factors (gender, age, and ISS stage) had a statistically significant impact on patient survival (Fig. 11A). The multivariate Cox regression analysis displayed that age, ISS stage, and RS were the independent prognostic factors (Fig. 11B). To construct a nomogram with the powerful predictive ability, we generated four nomograms with different variables and calculates the c-index values. The nomograms constructed by ISS stage, RS, and ISS stage combined with gender and age had a c-index of 0.661, 0.648, and 0.690, respectively, while the nomogram (Fig. 11C) constructed by gender, age, ISS stage, and RS had a c-index of 0.721, indicating the best prognostic predictive ability. The calibration curves of the nomogram with 4 variables predicting 1-, 3-, and 5-year survival for patients with MM were shown in Fig. 11D-F.

## Discussion

MM is the second most common hematological malignancy. Despite recent advances in therapy, there are still subsets of patients with poor survival. At present, lots of prognostic markers have been found. Whereas they are not sufficient to change the treatment of MM, and most patients still use the same treatment protocols. New approaches to better risk stratify patients with MM are necessary [28, 29]. Pyroptosis is an inflammatory cell death mediated by caspase and gasdermin family proteins; under the action of caspases, gasdermins can punch holes in the cell membrane, releasing inflammatory factors and inducing cell death [30]. In recent years, pyroptosis has received great attention due to its effect on antitumor immunity [31, 32]. Besides, several studies showed that pyroptosis could fuel tumor progression [33–35]. These suggested that pyroptosis might have potential prognostic value. However, there are still no exactly prognostic markers for pyroptosis in MM. To evaluate the role of PRGs in the prognosis of MM, we performed this study.

By comparing the expression of PRGs in the MM cells and normal plasma cells, 12 differentially expressed PRGs were identified. Consistent with the prior study [12], GSDMB and AIM2 were overexpressed in the MM group. Furthermore, their expression levels increased with the ISS stage. GSDMB, belonging to the gasdermin family, can trigger pyroptosis after cleavage [36]. It is highly expressed in a variety of tumors, and correlated with cancer cell invasion, progression, and metastasis [37]. AIM2 is a sensor molecule, which can directly recognize double-stranded DNA and form an activated inflammasome with apoptosis-associated speck-like protein containing a CARD (ASC) and caspase-1, thereby inducing pyroptosis [38]. High expression of AIM2 is observed in some malignancies, e.g., lung cancer, and nasopharyngeal carcinoma; depending on the type of cancer, AIM2 plays a pro-cancer or anti-cancer role [39]. The relationship between AIM2 and MM is unclear. By the immune infiltration analysis, we found it was significantly negatively correlated with activated NK cells, the reduction of which promotes the formation of an immunosuppressive microenvironment in MM [40]. The prognosis analysis revealed that high expression of AIM2 predicted poor survival in MM.

A total of 33 PRGs in the MMRF project were relatively conserved and stably expressed. Based on these genes, we defined 4 MM molecular subtypes and found that patients in cluster 1 had poorer survival than those in cluster 2. The activities of two differential pathways, including the toll-like receptor signaling pathway and cytosolic DNA sensing pathway, were upregulated in cluster 1, which can induce an immunosuppressive microenvironment and protect tumors from attack [41, 42]. The heatmap of PRG expression in 4 clusters indicated that IL-6 was overexpressed in cluster 1. IL-6 is a member of the pro-inflammatory cytokine family and plays an important role in mediating drug resistance and survival in MM [43]. The immune infiltration analysis revealed that the infiltration degrees of many immune cells were different in cluster 1 and cluster 2, which indicates the composition of the immunological microenvironment may affect the prognosis of myeloma. Li et al. [13] also performed a clustering analysis based on the TCGA-MMRF database and identified two clusters using the R package “ConsensusClusterPlus”. Similar to the findings of our study, their study showed that the levels of immune activation were different in two clusters, indicating that pyroptosis could be involved in defining the immunological microenvironment of MM [13].

To construct a prognostic model, 9 PRGs were screened out as prognostic markers, namely AIM2, CASP5, IL1B, IL6, NLRP6, NOD1, PRKACA, PYCARD, and SCAF11, the RS consisting of which had high predictive performance, and the nomogram showed the powerful predictive ability. Compared to the RS in the previous studies [12, 13], we found that AIM2 was present in all three studies, while IL-IB was present in our study and Wang et al.‘s study [12]. Li et al. [13] detected the relative expression of AIM2 in the MM group and the control group by quantitative real-time PCR and found there were no differences in the expression of AIM2 (*p* = 0.079), but the control samples they chose were from patients with iron deficiency anemia, and they didn’t describe whether they isolated normal plasma cells. IL1B is an important mediator of the inflammatory response and has a dual effect on tumors [44]. Takagi et al. [45] demonstrated that IL-1B is critical to platelet-mediated MM progression. For the remaining 7 genes, CASP5 is a member of the caspase family. After activation, it can cleave GSDMD to execute pyroptosis and stimulate inflammation [46]. NLRP6 is the sensor component of the NLRP6 inflammasome, which mediates the maturation and secretion of IL-18 and IL-1B [47]. Yu et al. [48] revealed that NLRP6 inflammasome interacted with SP1 to induce immune evasion in glioma cells. As with NLRP6, NOD1 is also a member of the NOD-Like Receptor (NLR), and it could modulate the immunosuppressive activity of myeloid cells in colorectal cancer [49]. PRKACA is one of the catalytic subunits of protein kinase A and was found to mediate resistance to HER2-targeted therapy in breast cancer [50]. PYCARD is an adaptor protein that assembles the inflammasome, high expression of which was considered to be an independent predictor of unfavorable prognoses in glioma and could promote glioma cell proliferation and migration [51]. SCAF11, also known as caspase-11, is a pro-inflammatory enzyme, which may have a role in cancer-associated angiogenesis [52]. Chu et al. [53] demonstrated that knocking down SCAF11 suppressed cell proliferation and colony formation in breast cancer cell lines.

There are still some limitations in the study. Firstly, the revised ISS (R-ISS) stage was not included in our predictive model because of the lack of related data in the MMRF project. Compared with the ISS stage, the R-ISS stage incorporates two additional prognostic factors: genomic features and LDH levels at diagnosis. It is now considered the standard risk stratification model for patients with newly diagnosed MM, although it classifies most patients into the intermediate-risk category (R-ISS II) [54]. Hence, our model may lack some predictive power. Then, no experiments were done to validate our results. We identified 12 differentially expressed PRGs and 9 PRGs as prognostic markers, which should be verified by western blot (WB), quantitative real-time PCR, and a clean loss-of-function and gain-of-function study. Besides, further clinical analysis is necessary to detect the prognostic performance of these genes.

In conclusion, we identified 9 PRGs as prognostic markers for MM and constructed a prognostic predictive model with high predictive performance. This model can enhance the predictive ability of ISS staging and give a reference for clinical decision-making. These new prognostic markers based on pyroptosis could provide some insights for follow-up studies and facilitate the development of novel risk stratification for MM.

## Electronic supplementary material

Below is the link to the electronic supplementary material.

Supplementary Material 1

Supplementary Material 2

## Data Availability

The datasets generated during and/or analyses during the current study are available in the TCGA and GEO Database.

## Abbreviations

ASC: apoptosis-associated speck-like protein containing a CARD
AUC: area under the curve
AIM2: absent in melanoma 2
BH: Benjamini-Hochberg
CASP1: cysteine-aspartic acid protease-1
CASP3: cysteine-aspartic acid protease-3
CASP4: cysteine-aspartic acid protease-4
CASP5: cysteine-aspartic acid protease-5
CASP6: cysteine-aspartic acid protease-6
CASP8: cysteine-aspartic acid protease-8
CASP9: cysteine-aspartic acid protease-9
C-index: consistency index
ELANE: elastase, neutrophil expressed
GEO: Gene Expression Omnibus
GPX4: glutathione peroxidase 4
GSDMA: gasdermin A
GSDMB: gasdermin B
GSDMC: gasdermin C
GSDMD: gasdermin D
GSDME: gasdermin E
GSVA: Gene set variation analysis
K-M: Kaplan-Meier
IL-18: interleukin 18
IL-1B: interleukin 1 beta
IL-6: interleukin 6
ISS: International Staging System
MM: multiple myeloma
NLR: NOD-Like Receptor
NLRC4: NLR family CARD domain containing 4
NLRP1: NLR family pyrin domain containing 1
NLRP2: NLR family pyrin domain containing 2
NLRP3: NLR family pyrin domain containing 3
NLRP6: NLR family pyrin domain containing 6
NLRP7: NLR family pyrin domain containing 7
NMF: Non-negative matrix factorization
NOD1: nucleotide binding oligomerization domain containing 1
NOD2: nucleotide binding oligomerization domain containing 2
PJVK: pejvakin/deafness, autosomal recessive 59
PLCG1: phospholipase C gamma 1
PRGs: pyroptosis-related genes
PRKACA: protein kinase cAMP-activated catalytic subunit alpha
PYCARD: PYD and CARD domain containing
R-ISS: revised ISS
RNA-seq: transcriptome sequencing
ROC: receiver operating characteristic
RS: risk score
SCAF11: SR-related CTD associated factor 11
TCGA: The Cancer Genome Atlas
TIRAP: TIR domain containing adaptor protein
TNF: tumor necrosis factor
WB: western blot
WES: exome sequencing
